# Thymoquinone Crosstalks with DR5 to Sensitize TRAIL Resistance and Stimulate ROS-Mediated Cancer Apoptosis

**DOI:** 10.31557/APJCP.2021.22.9.2855

**Published:** 2021-09

**Authors:** Ahmed A Abd-Rabou, Nagwa M Abd El-Salam, Hayat M I Sharada, Gehan G Abd EL Samea, Mohga S Abdalla

**Affiliations:** 1 *Department of Hormones, Medical Research Division, National Research Centre, Cairo, Egypt. *; 2 *Egyptian Company for Blood Transfusion Services (EgyBlood), 51 Wezaret El-Zeraa Street, VACSERA, Agouza, Giza, Egypt. *; 3 *Department of Chemistry, Faculty of Science, Helwan University, Egypt. *

**Keywords:** TRAIL, thymoquinone, DR5, apoptotic genes, breast cancer cell lines

## Abstract

**Objective:**

Cancer treatment using a targeted inducer of apoptosis like tumor necrosis factor-related apoptosis-inducing ligand (TRAIL) faced the obstacle of resistance, thus providing a plus drug like Thymoquinone (TQ) could be of great interest to tackle breast cancer cells. The aim of the present work is to examine the genetic modulation impacts of the TRAIL receptors and apoptotic markers upon the combinatorial remedy of TRAIL plus TQ on human breast cancer cell lines.

**Methods:**

To achieve this rationale, the protein content-based cytotoxicity using SRB assay, as well as the genetic expressions of the TRAIL receptors (DR4 and DR5) and apoptotic markers (Bcl-2, Cas-8, and FADD) using real time qRT-PCR technique were preceded against breast cancer MCF-7 and MDA-MB-231 cancerous cell lines.

**Results:**

The current study showed that the combination therapy of TQ+TRAIL significantly inhibited the protein content-based proliferation of MDA-MB-231 cells more than MCF-7 cells. The synergistic effect of them significantly up-regulated the genetic expressions of *DR4, DR5, Cas-8*, and *FADD* genes and inhibited the genetic expression of the *Bcl-2* gene in the proposed cell lines treated for 24 h. The induction of the apoptotic genes using the combined therapy was stimulated by the elevation of the reactive oxygen species (ROS); nitric oxide (NO) and malondialdehyde (MDA) levels.

**Conclusions:**

The synergistic influence between TQ which induced the DR5 and TRAIL, facilitating the connection between TRAIL and its receptors on the cancerous cell membrane. Hence, the proposed combination therapy induced the ROS-mediated apoptotic stimulus.

## Introduction

Breast cancer (BC) is the second leading cause of death in women, accounting for roughly 30% of new occurrences (Zeng et al., 2017). Breast, liver, and bladder cancers are the most common cancers in both men and women in Egypt, according to the publications archive. Females were more likely to have breast and hepatic cancers, while males were more likely to develop hepatic and bladder cancers (Kassem et al., 2019).

Human breast cancer MCF-7 and MDA-MB-231 cell lines provide suitable models for *in vitro* treatment of breast cancer. Ovarian estrogens are required for the onset and progression of human breast cancer. The MCF-7 breast cancer cell line possesses efficient oestrogen receptors (ER), but the MDA-MB-231 human breast cancer cell line lacks ER and is unresponsive to ER and anti-ER (Ren et al., 2018).

There are numerous community therapies for various malignancies that prevent cancer proliferation by interfering with particular particles that are beneficial to cancer growth. The selected treatments are based on a better understanding of molecular genetics in malignant cell progression, which is used in the development of desirable remedies (Mansoori et al., 2017). 

One of these crucial medicines, TNF-related apoptosis-inducing ligand (TRAIL) (Rieger et al., 1999), is marketed to specifically induce apoptosis in malignant cells such as breast, bladder, liver, and lung (Turner et al., 2014; Szliszka et al., 2012). TRAIL is a cytokine that is generated by various organs and has anticancer effect; hence it is found in the milk of nursing mothers (Zauli et al., 2013). TRAIL has been implicated in the eradication of cancer and the halting of its spread (Allen et al., 2013).

TRAIL can attach to its receptors (DR4 and DR5), triggering apoptosis through the intracellular death domain (DD) (Gasparian et al., 2009). The apoptotic signalling pathway produced by TRAIL is mediated by TRAIL binding to its receptors (DR4 and/or DR5), followed by receptor clustering, and then recruitment of the Fas-associated protein with death domain (FADD). The death-inducing signalling complex (DISC), also known as the 1^st^ complex, is formed when this FADD recruits caspase-8. This is followed by mitochondrial malfunction and the release of a mitochondrial activator (Smac) (Du et al., 2000).

Because TRAIL administration is difficult due to the development of TRAIL resistance, TRAIL combos have been proposed for over fifteen years to generate synergism or sensitise resistant cancer cells. Chemotherapy, which is either from natural agents or produced in the lab, limits the spread of cancer cells by preventing DNA damage that recruits cancer cells and generating apoptotic signals or preventing premalignant cells from proliferating into malignancy (Sun et al., 2017).

Many medicinal herbs have natural components that may be used to cure a hazardous and fatal disease such as carcinoma. The use of a natural agent with no side effects is critical in the treatment of such cancers (Rafieian-Kopaei et al., 2017). 

Thymoquinone (TQ), a potential cancer chemotherapeutic candidate derived from dietary components, has been demonstrated to have a significant role in overcoming the resistance of several malignancies, including breast and liver cancers, in multiple *in-vitro *and *in-vivo* studies (LIU et al., 2017; Woo et al., 2012). 

TQ, a key component of Nigella Sativa, has been demonstrated to have anticancer properties (Woo et al., 2012).

TQ can be utilised as an anti-proliferative, pro-apoptotic, anti-angiogenic, and anti-angiogenic agent, as well as a cell cycle arrest inducer, according to the molecular signals (Majdalawieh and Fayyad, 2016; ElKhoely et al., 2015). TQ induced apoptosis and inhibited growth in pancreatic cancer cells, according to previous research (Chehl et al., 2009). Others looked at whether TQ had an inhibitory effect on malignant cell growth in a variety of cell types (Chen et al., 2012). 

Overall, in the current study, we investigated the effect of TQ and TRAIL either individually or in combination as anticancer agents through studying the genetic expressions of the TRAIL receptors (DR4 and DR5) and the apoptotic signals (Bcl-2, Cas-8, and FADD genes). In addition, the reactive oxygen species (ROS); nitric oxide (NO) and malondialdehyde (MDA) levels were tested upon treatments on both cancerous cell lines (MCF-7 and MDA-MB-231).

## Materials and Methods

*Materials*

Thymoquinone was purchased from Santa Cruz Biotechnology. TNF-related apoptosis-inducing ligand (TRAIL) and sulforhodamine B (SRB) for cellular protein measurement was purchased from Sigma-Aldrich (St. Louis, MO, USA). Dulbecco’s Modified Eagle Medium (DMEM), Roswell Park Memorial Institute (RPMI) 1640 medium, fetal bovine serum (FBS), penicillin/streptomycin (P/S), l-glutamine, trypsin/EDTA, phosphate-buffered saline (PBS), and Annexin V stain for apoptosis analysis were obtained from Life Technologies, Gibco (Grand Island, NY, USA). Nitric oxide (NO) and malondialdehyde (MDA) were purchased from (BIODIAGNOSTIC, Egypt). RNA Purification kit and RevertAid First strand cDNA synthesis kit were purchased from (Thermo Fisher Scientific, USA). SensiFAST SYBR LO-ROX mix kits were purchased from (Sigma). Primers for FADD, Caspase 8, Bcl-2, DR4 and DR5 versus GAPDH as a house keeping gene were generated using Primer3 program and verified by NCBI/ Primer-BLAST.

*Cell Lines*

Human breast cancer MCF7 (Cat. No. HTB-22™) and MDA-MB-231 (Cat. No. HTB-26™) cell lines were purchased from Cell Culture Department, The Egyptian Holding Company for Biological products and vaccines (VACSERA), Giza, Egypt which purchased them from American Type Culture Collection (ATCC).

*In-vitro studies*

*Cell culture and maintenance*

MDA-MB-231 breast cancerous cell line is listed among the estrogen (ER), progesterone (PR), and HER2 triple-negative metastatic breast cancer panel (ATCC^® ^No. TCP-1002™). Despite both MDA-MB-231 and MCF7 cell lines are breast cancer cell lines, MDA-MB-231 is “basal” type and triple negative and MCF7 is “luminal” type and ER and PR positive cell lines. Thus, these differences affect the drug sensitivity. Breast cells were cultured using Dulbecco’s modified Eagle’s medium (DMEM) and Roswell Park Memorial Institute (RPMI-1640) medium. All media were supplemented with 4.5g/L Glucose with L-Glutamine and 10% fetal bovine serum (FBS). The cells were incubated in 5% CO_2_ humidified at 37°C for growth maintenance.

*Cellular protein measurements*

The protein contents of the human breast cancerous MCF-7 and MDA-MB-231 cell lines up on treatments with TQ, TRAIL, TQ+TRAIL, and 5-FU were measured according to the previously optimized method (Ahmed et al., 2015). Basically, sulforhodamine B (SRB), the protein dye, binds electrostatically and pH dependent on protein basic amino acid residues of trichloroacetic acid-fixed MCF-7 and MDA-MB-231 cells. After incubation with the proposed treatments, cell monolayers are fixed with 10% (wt/vol) trichloroacetic acid and stained with the SRB for 30 min, after which the excess dye is removed by washing repeatedly with 1% (vol/vol) acetic acid. The protein-bound dye is dissolved in 10 mM Tris base solution and observed at 510 nm using a microplate ELISA reader. This experiments were repeated three times (n=3) and the results represented a method for detection the cytotoxic effect on the proposed cancerous cells based on the cellular protein content. 

*The half inhibitory concentration (IC*_50_ *) of the cancerous cellular protein*

The half maximal inhibitory concentrations (IC_50_) values which means that the concentrations of the used TQ, TRAIL, TQ+TRAIL, or 5-FU inhibit 50% of cell viabilities-based cellular protein content using MCF-7 and MDA-MB-231 cell lines, were got by plotting the percentages of cell viabilities versus the concentrations of the sample using polynomial concentration–response curve fitting models (OriginPro 8 software). 

*Apoptosis assay*

Apoptosis analysis was performed after fluorescence labeling of the damaged cancerous MCF-7 and MDA-MB-231 cellular membrane with Annexin V stain after treatment with the IC_50_ dosage of the proposed TQ, TRAIL, TQ+TRAIL, or 5-FU. The apoptotic fluorescence analysis, using (BMG Labtech, Germany), was performed to test the apoptotic shift after TQ, TRAIL, TQ+TRAIL, or 5-FU against control. Finally, the apoptotic cells (%) of the MCF-7 and MDA-MB-231 breast cancerous cells were analyzed and figured out.

*Measurements of stress markers*

Nitric oxide (NO): NO plays an important role in trigging cancerous cell apoptosis. NO is rapidly oxidized to nitrite and nitrate which are used to quantitate NO production. Briefly, the MCF-7 and MDA-MB-231 cells were cultured in 96-well plates at a density of 1×10^4^ cells/well. In the second day, the IC_50_ dosage of the proposed TQ, TRAIL, TQ+TRAIL, or 5-FU was added in the media. Nitrate reductase was firstly used to converts nitrate to nitrite. Then, Griess reagent was used to converts nitrite to a deep purple azo compound. The amount of the azo chromophore accurately reflected NO amount in the samples. Finally, optical density was measured at OD 540 nm using the microplate reader (BMG Labtech, Germany).

Malondialdehyde (MDA): Lipid peroxidation refers to the oxidative degradation of the cellular membrane lipids (to confirm annexin V-based apoptosis). In this process free radicals take electrons from the lipids in cell membranes, resulting in MCF-7 and MDA-MB-231 cells damage. The end product of lipid peroxidation is MDA. Briefly, the cells were cultured in 96-well plates at a density of 1×10^4^ cells/well. In the second day, the IC_50_ dosage of the proposed TQ, TRAIL, TQ+TRAIL, or 5-FU was added in the media. The free MDA present in the sample was reacted with Thiobarbituric Acid (TBA) to generate a MDA-TBA adduct, which can be easily quantified colorimetrically at OD 532 nm using the microplate reader (BMG Labtech, Germany).

*Real Time qRT-PCR*

Total RNA Extraction: Total RNA was extracted from untreated control cells as well as treated MCF-7 and MDA-MB-231cells with the IC_50_ dosage of the proposed TQ, TRAIL, TQ+TRAIL, or 5-FU using the Invitrogen RNA Purification kit (Thermo Fisher) according to the manufacturer’s protocol. The concentration and the purity of RNA were assessed by Nanodrop Technologies at 260/280 ratio.

Conversion of RNA to cDNA: First-strand cDNA was synthesized with 1 µg of total RNA using a RevertAid First strand cDNA synthesis kit (Thermo Fisher Scientific, USA) in accordance with the manufacturer’s instructions. These samples were subsequently frozen at a temperature of -80^o^C until use for determination of the expression levels of (*FADD, Caspase 8, Bcl-2, DR4* and *DR5*) genes using real-time PCR. 

*Real Time PCR reactions*

Quantitative real-time PCR was performed on Stratagene (Agilent) Mx3005P™ using the SensiFAST SYBR LO-ROX mix kits in addition to the forward and reverse primers for each gene. The nucleic acid sequences of the primers were as follows: Bcl-2 (forward: 5’-CTGCACCTGACGCCCTTCACC-3’& reverse: 5’-CACATGACCCCACCGAACTCAAAGA-3’), caspase8 (forward: 5’- AGAGTCTGTGCCCAAATCAAC-3’& reverse 5’- GCTGCTTCTCTCTTTGCTGAA- 3’), FADD (forward: 5’- CTCAGGTCCTGCCAGATGAAC-3’ & reverse: 5’-GGACGCTTCGGAGGTAGATG-3’), DR4 (forward: 5’ -TCCAGCAAATGGTGCTGAC-3’ & reverse: 5’-GAGTCAAAGGGCACGATGTT-3’), DR5 (forward: 5’-CCAGCAAATGAAGGTGATCC-3’& reverse: 5’-GCACCAAGTCTGCAAAGTCA-3’) compared to *β-actin* as a housekeeping gene (forward: 5’-TTGCCGACAGGATGCAGAA-3’ & reverse: 5′- GCCGATCCACACGGAGTACT -3’). 

Real-time PCR mixture consisted of 10 µL 2x SensiFAST SYBR LO-ROX PCR Master Mix, 1 µL of each primer (400nM ), 1 µL cDNA, and 7 µL Rnase-free water in a total volume of 20 µL. Amplification conditions and cycle counts were a temperature of 95^o^C for 15 min for the initial activation, followed by 40 cycles of denaturation at 95^o^C for 30 s, annealing at 55^o^C for 30 s, and elongation at 72^o^C for 30 s. Melting curves were performed after real-time PCR to demonstrate the specific amplification of single products of interest. A standard curve assay was performed to determine the amplification efficiency of the primers used. Relative fold changes in the expression of target genes were accomplished using the comparative 2^−ΔΔCt^ with the* β-actin *gene as an internal control to normalize the level of target gene expression. ΔΔCT is the difference between the mean ΔCT (treatment group) and mean ΔCT (control group), where ΔCT is the difference between the mean *CT* gene of interest and the mean CT internal control gene in each sample. Logarithmic transformation was performed on fold change values before being statistically analyzed, using the fold change values of three replicates for each gene measured. 

*Statistical analysis*

All experiments were carried out in three independent tests (n=3). Data were expressed as the mean ± standard deviation (SD) and analyzed using one-way analysis of variance (ANOVA). The results were considered statistically significant at probability < 0.05.

## Results

*Cellular protein-based cytotoxicity and the IC*_50_

The purpose of this study was to use the SRB assay to assess the cellular protein content of MCF-7 and MDA-MB-231 cells. As a combinatorial anticancer drug, this reflects the cytotoxic action of TRAIL and TQ. The anticancer capability of TRAIL and/or TQ against human breast cancer cell lines reflects the cells’ normal function, and so cytotoxicity and cell viability are measured. 

We initially studied the effect of TQ on the breast cancer cell death to investigate the effects of TQ on cancer cell growth, which showed that TQ can decrease the cell growth of both MDA-MB-231 and MCF-7 cancerous cells (Tables 1, 2). Using 5-Flurouracil (5-FU) as a positive control anti-cancer drug, the used 5-FU concentrations in the study were as follows: 0 (untreated cells), 20, 40, 60, 80, 100 μM in parallel with the proposed TQ in μM unit, but the concentrations of TRAIL were the same but in nM unit.

There were a gradual and significant decrease (p less than 0.05) the cancerous cell viability using SRB in both MCF-7 and MDA-MB-231 cancerous cells upon TQ treatment. In details, there was a slight cellular protein reduction effect of TQ on MCF-7 breast cancerous cells at 20 μM, where the cell viability was 82.816± 4.14 % after 24 h. We found significant reduction effects of TQ upon MCF-7 breast cancerous cells until reached 47.331± 2.36 % cell viability at 100 μM after 24 h (Table 1), with the half maximal inhibitory doses (IC_50_) value of TQ upon MCF-7 breast cancerous cells equal 69.306± 3.46 μM (Table 3).

For MDA-MB-231 cancerous cells, there was a slight cellular protein reduction effect of TQ at 20 μM, where the cell viability was 60.124± 3.00 % after 24 h. We found significant reduction effects of TQ upon MDA-MB-231 breast cancerous cells until reached 46.563± 2.32 % cell viability at 100 μM after 24 h (Table 2), with the half maximal inhibitory concentrations (IC_50_) value of TQ upon MDA-MB-231 breast cancerous cells equal 54.327± 2.71 μM (Table 3).

There were a gradual and significant decrease (p less than 0.05) the cancerous cell viability using SRB in both MCF-7 and MDA-MB-231 cancerous cells upon TRAIL treatment. In details, there was a slight cellular protein reduction effect of TRAIL on MCF-7 breast cancerous cells at 20 μM, where the cell viability was 83.505± 4.17 % after 24 h. We found significant reduction effects of TRAIL upon MCF-7 breast cancerous cells until reached 47.337± 2.36 % cell viability at 100 μM after 24 h (Table 1), with the half maximal inhibitory concentrations (IC_50_) value of TRAIL upon MCF-7 breast cancerous cells equal 67.489± 3.37 μM (Table 3).

For MDA-MB-231 cancerous cells, there was a slight cellular protein reduction effect of TRAIL at 20 μM, where the cell viability was 85.140± 4.25 % after 24 h. We found significant reduction effects of TRAIL upon MDA-MB-231 breast cancerous cells until reached 21.063± 1.05 % cell viability at 100 μM after 24 h (Table 2), with the half maximal inhibitory concentrations (IC_50_) value of TRAIL upon MDA-MB-231 breast cancerous cells equal 68.090± 3.40 μM (Table 3).

There were a gradual and highly significant decrease (p less than 0.01) the cancerous cell viability using SRB in both MCF-7 and MDA-MB-231 cancerous cells upon TQ+TRAIL treatment. In details, there was a slight cellular protein reduction effect of TQ+TRAIL on MCF-7 breast cancerous cells at 20 μM, where the cell viability was 52.163± 2.60 % after 24 h. We found significant reduction effects of TQ+TRAIL upon MCF-7 breast cancerous cells until reached 13.714± 0.68 % cell viability at 100 μM after 24 h (Table 1), with the half maximal inhibitory concentrations (IC_50_) value of TQ+TRAIL upon MCF-7 breast cancerous cells equal 26.045± 1.30 μM (Table 3).

For MDA-MB-231 cancerous cells, there was a slight cellular protein reduction effect of TQ+TRAIL at 20 μM, where the cell viability was 43.517± 2.17 % after 24 h. We found significant reduction effects of TQ+TRAIL upon MDA-MB-231 breast cancerous cells until reached 1.963± 0.09 % cell viability at 100 μM after 24 h (Table 2), with the half maximal inhibitory concentrations (IC_50_) value of TQ+TRAIL upon MDA-MB-231 breast cancerous cells equal 25.171± 1.25 μM (Table 3).

For 5-FU positive anti-cancer drug, there were a gradual and significant decrease (p less than 0.05) the cancerous cell viability using SRB in both MCF-7 and MDA-MB-231 cancerous cells upon 5-FU treatment. In details, there was a slight cellular protein reduction effect of 5-FU on MCF-7 breast cancerous cells at 20 μM, where the cell viability was 92.791± 4.64 % after 24 h. We found significant reduction effects of 5-FU upon MCF-7 breast cancerous cells until reached 46.337± 2.31 % cell viability at 100 μM after 24 h (Table 1), with the half maximal inhibitory concentrations (IC_50_) value of 5-FU upon MCF-7 breast cancerous cells equal 92.231± 4.61 μM (Table 3).

For MDA-MB-231 cancerous cells, there was a slight cellular protein reduction effect of 5-FU at 20 μM, where the cell viability was 100.459± 5.02 % after 24 h. We found significant reduction effects of 5-FU upon MDA-MB-231 breast cancerous cells until reached 44.763± 2.23 % cell viability at 100 μM after 24 h (Table 2), with the half maximal inhibitory concentrations (IC_50_) value of 5-FU upon MDA-MB-231 breast cancerous cells equal 92.439± 4.62 μM (Table 3).

*TRAIL-receptor genes*

In the current study, we showed that the TQ sensitized TRAIL-mediated apoptosis via up-regulation of TARIL receptors, especially DR5. These results provide the evidence that combined treatment with TQ may be a novel therapeutic approach for the successful TRAIL-based cancer.

**Figure 1:**
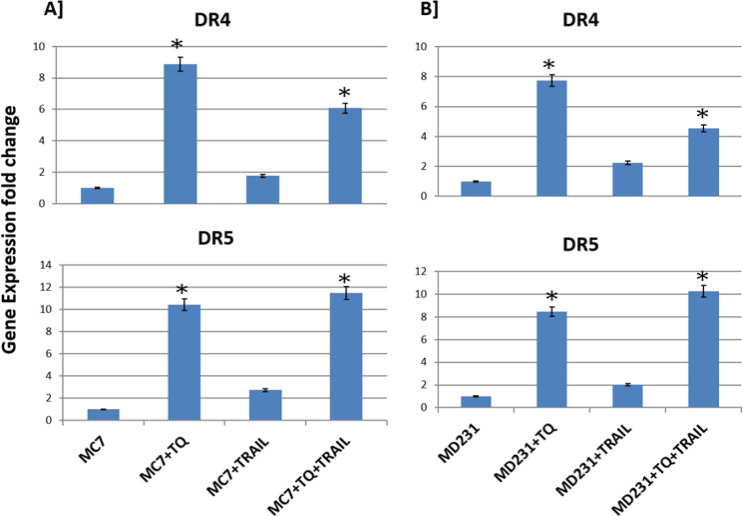
Genetic Expressions of the TRAIL Receptors. The individual TQ was firstly used to significantly upregulate the expressions of TRAIL-receptors, especially DR5, allowing the TRAIL subsequently to bind its DR receptors on the cancerous cell membranes, interfering its expression, so that the TRAIL receptors were not significantly upregulated. Collectively, both TQ+ TRAIL significantly induce the upregulation of TRAIL receptors, especially the DR5 TRAIL receptor. *; means high significance (p less than 0.01) when comparing treated cells with untreated cells

Regarding TRAIL-receptors story, the individual TQ was firstly used to significantly upregulate the expressions of TRAIL-receptors, especially DR5, allowing the TRAIL subsequently to bind its DR receptors on the cancerous cell membranes, interfering its expression, so that the TRAIL receptors were not significantly upregulated. Collectively, both TQ+TRAIL significantly induce the upregulation of TRAIL receptors, especially the DR5 TRAIL receptor. These results happened up on treatment of breast cancer MCF-7 and MDA-MB-231 cell lines incubated for 24 h with noted TQ and/or TRAIL concentrations. The genetic levels of *DR4* and *DR5* genes were quantiﬁed using real time qRT-PCR and normalized to *β-actin* housekeeping gene (Figure 1).

TQ increased the expression of TRAIL receptors, although at different potencies. TQ was relatively more potent inducer of TRAIL-R2 (DR5) than TRAIL-R1 (DR4) (Figure 1). However, both death receptors were up-regulated by the TQ. This clearly suggests that TRAIL receptors, at least partly, contribute to TQ-induced MDA-MB-231 and MCF-7 cell inhibition by likely activating the extrinsic pathway of apoptosis in TQ-treated MDA-MB-231 and MCF-7 cell lines. 

These results happened up on treatment of breast cancer MCF-7 and MDA-MB-231 cell lines incubated for 24 h with noted TQ and/or TRAIL concentrations. The genetic levels of the *DR4* and *DR5* genes were quantiﬁed using qRT-PCR and normalized to *β-actin* housekeeping gene.

*Apoptosis shifting*

**Figure 2:**
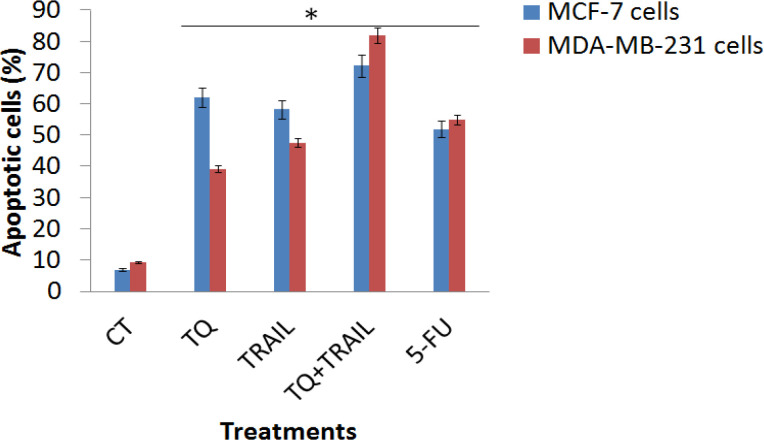
Apoptotic Cancerous MCF-7 and MDA-MB-231 Cells (%). The apoptotic positive shifts of the apoptotic cancerous MCF-7 and MDA-MB-231 cells (%) were showed upon the proposed treatment, with the highest significance when using TQ+TRAIL combination therapy (p less than 0.01). *; means high significance (p less than 0.01) when comparing treated cells with control

We next determined the mechanism by which TQ and TRAIL kill MCF-7 and MDA-MB-231 breast cancerous cell lines. We observed the highest significant enhancement of apoptosis (p less than 0.01) of MDA-MB-231 cells treated with TQ+TRAIL (82.0 % apoptosis versus 9.45 % of the untreated control) compared with the individual treatments with TRAIL and TQ (Figure 2). In MCF-7 cell line, we found that the combination therapy of TQ+TRAIL induced 72.3 % apoptosis versus 7.0 % of the untreated control. 

In addition, it was found that the individual treatments with TQ and TRAIL in MCF-7 and MDA-MB-231 breast cancerous cell lines represented a lower significant increase in apoptosis compared to the combinational therapy of them. Intriguingly, the apoptosis was induced more in MCF-7 breast cancerous cell line using TQ and TRAIL compared to MDA-MB-231 breast cancerous cell line (Figure 2).

For the standard anti-cancer 5-FU treatment, its induction of apoptosis was significantly less than our combinational therapy with TQ+TRAIL, where it we observed a significant enhancement of apoptosis (p less than 0.01) of MDA-MB-231 cells treated with 5-FU (55.0 % apoptosis versus 9.45 % of the untreated control) compared with the individual treatments with TRAIL and TQ (Figure 2). In MCF-7 cell line, we found that the combination therapy of 5-FU induced 52 % apoptosis versus 7.0 % of the untreated control. 

Overall, the apoptotic positive shifts of the apoptotic cancerous MCF-7 and MDA-MB-231 cells (%) were showed upon the proposed treatment, with the highest significance with TQ+TRAIL combination therapy (p less than 0.01).

*TQ+TRAIL induce NO/MDA homeostasis*

**Figure 3:**
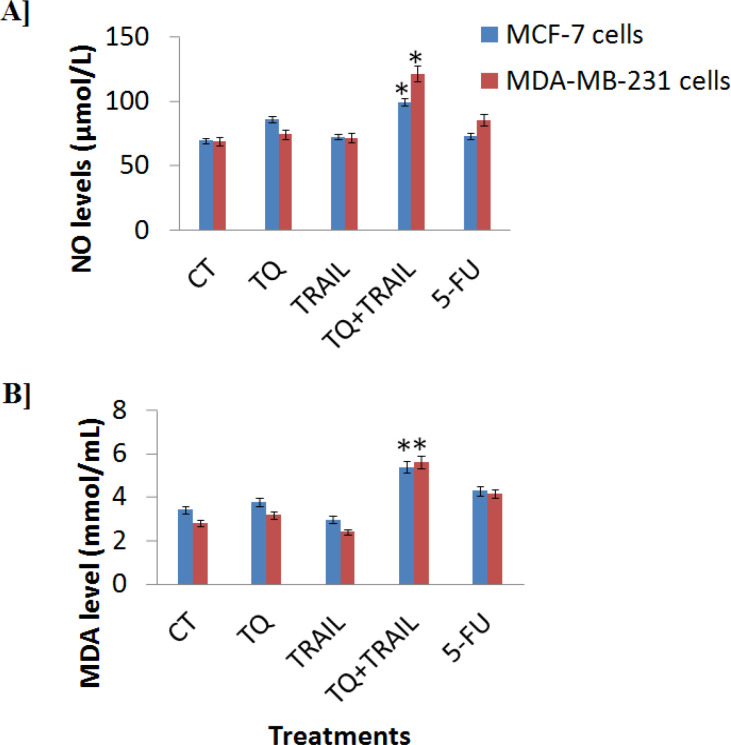
Stress Markers Levels in the Cancerous MCF-7 and MDA-MB-231 Cells. A] Nitric oxide NO levels were measured upon treatments with TQ, TRAIL, TQ+TRAIL, and 5-FU. B] Malondialdehyde MDA levels were measured upon treatments with TQ, TRAIL, TQ+TRAIL, and 5-FU. The combination therapy of TQ+TRAIL showed the highest significance* of the NO and MDA levels (p less than 0.01)

Fig. 3 shows the NO (Figure 3A) and MDA (Figure 3B) levels produced by MCF-7 and MDA-MB-231 cells up on treatments with TQ, TRAIL, TQ+TRAIL, and 5-FU versus control. Both oxidative stress markers (NO and MDA) were high significantly increased (P less than 0.01) up on the combinational treatment with TQ+TRAIL compared to the untreated control, while MDA was slightly abolished up on MCF-7 and MDA-MB-231 cells treatment with TRAIL alone. Then, their levels were lucidity statistical elevated (P less than 0.01) up on its combination with TQ compared to control. Intriguingly, the combination of both TQ+TRAIL re-balance the MDA and NO levels in MCF-7 and MDA-MB-231 cells, making this therapeutic combinatorial formula specific to kill breast cancer MCF-7 and MDA-MB-231 cells. On the other hand, the standard anti-cancer 5-FU agent induced also the oxidative stress markers (NO and MDA) in both cancerous cell lines, but in a minor effect (P less than 0.05) compared to our combinatorial therapy with TQ+TRAIL (P less than 0.01).

*Apoptotic Genes*

Total RNA was extracted from control, treated MCF-7 and MDA-MB-231 cells. The concentration and the purity of RNA were assessed by Nano drop Technologies at 260/280 ratio and by gel electrophoresis, respectively.

Relative fold changes in the expression of target genes were accomplished using the comparative 2^−ΔΔCt ^with the *β-actin* gene as an internal control to normalize the level of target gene expression. ΔΔCT is the difference between the mean ΔCT (treatment group) and mean ΔCT (control group), where ΔCT is the difference between the mean CT gene of interest and the mean *CT* internal control gene in each sample. Logarithmic transformation was performed on fold change values before being statistically analysed, using the fold change values of three replicates for each gene measured. 

**Figure 4:**
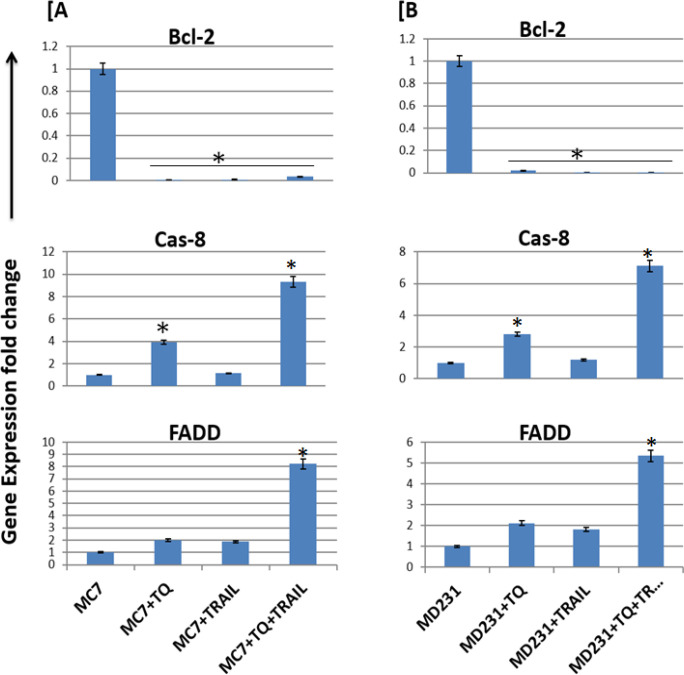
Genetic Expressions of the Apoptotic Genes. TQ and/or TRAIL inhibit the genetic expressions of *Bcl-2* gene and upregulate the genetic expressions of *Cas-8* and *FADD* genes up on breast cancer MCF-7 (A) and MDA-MB-231 (B) cell lines treated for 24 h with noted TQ and/or TRAIL concentrations. The amounts of* Bcl-2, Cas-8* and *FADD *genes were quantiﬁed using qRT-PCR and normalized to β-actin housekeeping gene. *; means high significance (p less than 0.01) when comparing treated cells with untreated cells

We examined whether TQ regulated expression of the anti-apoptotic gene *Bcl-2*. Real time Q-PCR analysis showed that TQ treatment at the IC_50_ for 24 h down-regulated mRNA level expression of the anti-apoptotic *Bcl-2 *gene in MDA-MB-231 and MCF-7 cells. TQ treatments caused decreased the expression of Bcl-2 (Figure 4), indicating TQ-induced MDA-MB-231 and MCF-7 cells growth suppersion. Our RT-PCR results intriguingly revealed that TQ enhanced RNA levels of the pro-apoptotic genes *Cas-8* and *FADD* (Figure 4), both effects are known to target cells to apoptosis, would suggest that even in the presence of antagonistic effect of Bcl2 increase, the net effect of TQ presence is likely increased mitochondrial, intrinsic apoptosis in the MDA-MB-231 and MCF-7 cells. 

The synergistic effect of the combination therapy of TQ and TRAIL significantly inhibit the genetic expressions of *Bcl2* gene and upregulate the genetic expressions of *Cas-8* and *FADD* genes up on breast cancer MCF-7 and MDA-MB-231 cell lines treated for 24 h with noted TQ or/and TRAIL concentrations. The amounts of *Bcl2, Cas-8 *and *FADD* genes were quantiﬁed in triplicate (n=3) using qRT-PCR and normalized to *β-actin* housekeeping gene (Figure 4).

**Table 1:** MCF-7 Cellular Protein Measurements Using SRB Assay

Treatment	TQ	TRAIL	TQ+TRAIL	5-FU
Conc µM, nM	Mean±SD	Mean±SD	Mean±SD	Mean±SD
0 (Control)	100±5	100±5	100±5	100±5
20	82.81±4.14	83.50±4.17	52.16±2.60	92.79±4.64
40	57.90±2.89	58.59±2.93	27.25±1.36	67.88±3.39
60	55.42±2.77	53.91±2.69	25.18±1.25	65.81±3.29
80	49.29±2.46	48.33±2.41	16.99±0.85	56.54±2.82
100	47.33±2.36	47.33±2.36	13.71±0.68	46.33±2.31

cTQ, Thymoquinone; 5-FU, 5-Flurouracil; Control, untreated cancerous MCF-7 cells; SRB, sulforhodamine B; SD, standard deviation

**Table 2:** MDA-MB-231 Cellular Protein Measurements Using SRB Aassay

Treatment	TQ	TRAIL	TQ+TRAIL	5-FU
Conc µM, nM	Mean±SD	Mean±SD	Mean±SD	Mean±SD
0 (Control)	100±5	100±5	100±5	100±5
20	60.12±3.00	85.14±4.25	43.51±2.17	100.45±5.02
40	55.15±2.75	74.86±3.74	28.15±1.40	75.32±3.76
60	55.88±2.79	55.95±2.79	25.29±1.26	75.51±3.77
80	48.72±2.43	39.06±1.95	10.94±0.54	57.54±2.87
100	46.56±2.32	21.06±1.05	1.96±0.09	44.76±2.23

TQ, Thymoquinone; 5-FU, 5-Flurouracil; Control, untreated cancerous MDA-MB-231 cells; SRB, sulforhodamine B; SD, standard deviation

**Table 3:** The IC50 of the Cellular Protein of MCF-7 and MDA-MB-231 Cell Lines

Treatments	TQ	TRAIL	TQ+TRAIL	5-FU
Cell lines	Mean±SD	Mean±SD	Mean±SD	Mean±SD
MCF-7	69.30±3.465	67.48±3.374	26.04±1.302	92.23±4.61
MDA-MB-231	54.32±2.716	68.09±3.405	25.17±1.259	92.43±4.62

TQ, Thymoquinone; 5-FU, 5-Flurouracil; SD, standard deviation; IC50, The half maximal inhibitory concentrations values which means that the concentrations of the used TQ, TRAIL, TQ+TRAIL, or 5-FU inhibit 50% of cell viabilities-based cellular protein content using MCF-7 and MDA-MB-231 cell lines.

## Discussion

The scarcity of death receptors expressed on the cell surface, and hence the lack of efficient targeting of these cells by TRAIL/agonistic mAb, limited the TRAIL-induced apoptosis method. Furthermore, the emergence of TRAIL resistance has unfavourable consequences for TRAIL therapy (Mahalingam et al., 2009). However, the second mitochondrial activator of caspases, Smac, which is produced from mitochondria during the development of pro-apoptosis, can counteract this impact (Kaufmann et al., 2012).

Faced with the development of resistance to TRAIL-targeted cell death, a new strategy has been devised in which TRAIL is paired with other medications (such as Thymoquinone TQ) that are more effective than TRAIL alone. Thymoquinone (TQ) is a potential natural chemical with considerable anticancer activity in vitro and in vivo against a variety of malignant cell types (Sarmistha et al., 2018). It is a potent inducer of cell cycle arrest and apoptosis. Preclinical studies reveal the potential of TQ in improving the therapeutic effect of anticancer drugs and also protection of non-tumor tissues against chemotherapy-induced damages (El-Baba et al., 2014).

The primary goal of combinatorial-based TRAIL is to either synergize TRAIL activity or sensitise TRAIL-resistant cells to TRAIL. To that purpose, most combinatorial techniques aimed at synergizing TRAIL and/or sensitising resistant tumours to TRAIL include chemical and natural substances (Zaidi et al., 2009). 

In parallel with the current study, the combination therapy of TQ+TRAIL had a gradual and highly significant decrease upon the cancerous protein content (i.e. cell viability) using SRB in both MCF-7 and MDA-MB-231 cells. The synergistic effect of this combinatorial therapy reported an IC_50_ value upon MCF-7 breast cancerous cells equal 26.045± 1.30 μM and an IC_50_ value upon MDA-MB-231 breast cancerous cells equal 25.171± 1.25 μM. 

Intriguingly, the combination therapy of TQ+TRAIL reported lower IC_50_ values compared to the standard 5-FU positive anti-cancer drug, which recorded an IC_50_ value upon MCF-7 cells equal 92.231± 4.61 μM and an IC_50 _value upon MDA-MB-231 breast cells equal 92.439± 4.62 μM. These findings validated the promising anticancer effect of TQ+TRAIL in breast cancer MCF-7 and MDA-MB-231 cells based on protein content (SRB test), which is consistent with our recently published paper that found similar results but used mitochondrial MTT labelling instead of SRB staining (Abd El-Salam et al., 2021). 

In the Middle East, thymoquinone, a bioactive component of Nigella sativa, is widely used (Badary et al., 2001). It contains antioxidant properties and has been found in animal experiments to protect against heart, liver, and kidney damage (Badary et al., 1997). TQ has been shown to decrease cell proliferation in a variety of cancer cells, including breast, colon, and lung cancer cells, in recent research. Previous research has demonstrated that TQ’s cytotoxic effects are limited to cancer cells (Banerjee et al., 2010). We reviewed the effects of TQ on the growth of breast cancer MCF-7 and MDA-MB-231 cells, as well as the genetic signals of TRAIL receptors (*DR4* and* DR5*) and apoptotic genes. In the case of TRAIL-receptors, the chemotherapy TQ was used to significantly upregulate the expressions of TRAIL-receptors, particularly DR5, allowing TRAIL to bind to its DR receptors on cancerous cell membranes, interfering with their expression, resulting in the TRAIL receptors not being significantly upregulated. Both TQ and TRAIL together dramatically increase TRAIL receptor expression. 

These findings came from the treatment of MCF-7 and MDA-MB-231 breast cancer cell lines. Combinatorial methods mostly work via endoplasmic reticulum (ER) stress, resulting in overexpression of DR5 and/or DR4 and increased TRAIL-induced apoptosis (Refaat et al., 2014). As a result, we examined stress markers (ROS) and discovered that the combined therapy with TQ+TRAIL remarkably induced the NO and MDA levels in the breast cancerous cells. 

The activation of CHOP via p38/ERK MAPKs, which increases DR5 transcription, is another downstream checkpoint (Woo et al., 2013). Up-regulating pro-apoptotic genes (Ghosh et al., 2012) or downregulating anti-apoptotic genes (Martn-Pérez et al., 2012), both of which are in perfect agreement with the current study’s findings. 

These previous results were in parallel with our results regarding the *DR4* and *DR5* genetic expression levels after the combination of TRAIL and TQ treatment, where in the current study, we showed that the TQ sensitizes TRAIL-mediated apoptosis via up-regulation of DR4 and DR5 as well as the enrolment of the ROS markers (NO and MDA). These results provide the evidence that combined treatment with TQ may be a novel therapeutic approach for the successful TRAIL-based cancer therapy.

To further elucidate molecular mechanisms of apoptosis signalling by TQ, we next determined alterations in levels of pro- and anti-apoptotic genes using qRT-PCR. Speciﬁcally, we examined expression of the pro-apoptotic genes and TRAIL receptors in TQ-treated breast cancer cell lines, MDA-MB-231 and MCF7 cells. TQ increased the expression of TRAIL receptors, although at different potencies. TQ was relatively more potent inducer of TRAIL-R2 (DR5) than TRAIL-R1 (DR4), as it up-regulated the expression of DR5, while DR4 expression was slightly affected. However, both death receptors were up-regulated by higher TQ concentrations. This clearly suggests that TRAIL receptors, at least partly, contribute to TQ-induced breast cancerous cell growth inhibition by likely activating the extrinsic pathway of apoptosis in TQ-treated cells. Our data support recent ﬁndings showing that DR5 (TRAIL-R2) is selectively up-regulated by TQ in human cancer cell lines (Hussain et al., 2011). Interestingly, Choi and companions (Choi et al., 2002) reported that DR5 ligation by TRAIL not only leads to apoptosis of human glioma cells but also induces expression of IL-8. Therefore, the TQ and TRAIL not only induce cancer cell death mediated by DR5 but also induce immune cell mediation.

The apoptotic ligand TRAIL binds to death domain-containing cell surface receptors, resulting in a connection between TRAIL receptors (*DR4* and *DR5*) and apoptotic genes (Rahman et al., 2009). Despite promising data indicating that TRAIL produced apoptosis in a large number of malignant cells, about half of cancer cell cultures were resistant to TRAIL-mediated cell death (Singh et al., 2003), as we reported in our study. Remarkably, up-regulation of DR4, DR5 and pro-apoptotic genes, as well as down-regulation of the anti-apoptotic B-cell lymphoma 2 (*Bcl-2*) gene and inhibitors of apoptosis in TRAIL-resistant tumor cell lines correlated with sensitization to TRAIL-induced apoptosis (Singh et al., 2003; Poulain et al., 2009), which in-line with our genetic profiling results.

TQ treatment for 24 hours reduced the mRNA level expression of the anti-apoptotic B-cell lymphoma 2 (*Bcl-2*) gene in MDA-MB-231 and MCF-7 breast cancer cells, according to real-time qRT-PCR analysis. These findings suggested that TQ inhibited the proliferation of MDA-MB-231 and MCF-7 cells. TQ’s downregulation of Bcl-2 is accompanied by an increase in DR5 (Gali Muhtasib et al., 2004; Liet al., 2011; Vikhanskaya et al., 2007).

The real time qRT-PCR results intriguingly revealed that the combination therapy of TQ+TRAIL significantly decreased the expression of the anti-apoptotic gene *Bcl-2* and increased expression of the pro-apoptotic genes *Cas-8 *and *FADD*, both effects are known to target cells to apoptosis, the net effect of the combination therapy is increased the mitochondrial, intrinsic apoptosis in the MDA-MB-231 and MCF-7cells as in discussed in previous publications (Abd El-Salam et al., 2021; Oltvai et al., 1993; LaCasse et al., 1998). 

Collectively, we may conclude that the combination of TRAIL and TQ have highly increased expression of the pro-apoptotic genes *Cas-8* and *FADD*, both effects are known to target cells to higher apoptosis mediated by the increment of *DR5* gene and *ROS* (NO and MDA) levels.

## Author Contribution Statement

All authors contributed in this work. HMI Sharada, GG Abd EL Samea, MS Abdalla, and AA Abd-Rabou are the supervisors of the NM Abdel Salam’s PhD thesis. AA Abd-Rabou had the work idea and was responsible for paper writing, editing, and publishing it, with the help of NM Abdel Salam. NM Abdel Salam and AA Abd-Rabou performed the cell culture, biochemical, and genetic experiments. HMI Sharada, GG Abd EL Samea, and MS Abdalla shared in all experiments and edited the manuscript.
